# Why did the buffalo cross the park? Resource shortages, but not infections, drive dispersal in female African buffalo (*Syncerus caffer*)

**DOI:** 10.1002/ece3.5145

**Published:** 2019-04-30

**Authors:** Robert S. Spaan, Clinton W. Epps, Vanessa O. Ezenwa, Anna E. Jolles

**Affiliations:** ^1^ Department of Fisheries and Wildlife Oregon State University Corvallis Oregon; ^2^ Department of Infectious Diseases, Odum School of Ecology University of Georgia Athens Georgia; ^3^ Department of Biomedical Sciences Oregon State University Corvallis Oregon; ^4^ Department of Integrative Biology Oregon State University Corvallis Oregon

**Keywords:** African buffalo, bovine tuberculosis, density dependence, disease, dispersal, gastrointestinal parasites, longitudinal study, resource limitation

## Abstract

Dispersal facilitates population health and maintains resilience in species via gene flow. Adult dispersal occurs in some species, is often facultative, and is poorly understood, but has important management implications, particularly with respect to disease spread. Although the role of adult dispersal in spreading disease has been documented, the potential influence of disease on dispersal has received little attention. African buffalo (*Syncerus caffer*) are wide‐ranging and harbor many pathogens that can affect nearby livestock. Dispersal of adult buffalo has been described, but ecological and social drivers of buffalo dispersal are poorly understood. We investigated drivers of adult buffalo dispersal during a 4‐year longitudinal study at Kruger National Park, South Africa. We monitored the spatial movement of 304 female buffalo in two focal areas using satellite and radio collars, capturing each buffalo every 6 months to assess animal traits and disease status. We used generalized linear mixed models to determine whether likelihood of dispersal for individual female buffalo was influenced by animal traits, herd identity, environmental variables, gastrointestinal parasites, or microparasite infections. The likelihood and drivers of buffalo dispersal varied by herd, area, and year. In the Lower Sabie herd, where resources were abundant, younger individuals were more likely to disperse, with most dispersal occurring in the early wet season and during an unusually dry year, 2009. In the resource‐poor Crocodile Bridge area, buffalo in poor condition were most likely to disperse. Our findings suggest that dispersal of female buffalo is driven by either seasonal (Lower Sabie) or perhaps social (Crocodile Bridge) resource restriction, indicating resource limitation and dispersal decisions are tightly linked for this social ungulate. We found no direct effects of infections on buffalo dispersal, assuaging fears that highly infectious individuals might be more prone to dispersing, which could accelerate the spatial spread of infectious diseases.

## 1 INTRODUCTION

Dispersal is an essential life‐history trait in animals, which functions as a mechanism for avoiding inbreeding and finding usable habitats (Bowler & Benton, 2005; Clobert, Danchin, Dhondt, & Nichols, 2001; Kokko & López‐Supulcre, 2006). Most studies of dispersal have explored natal dispersal, which is typically a permanent movement of juveniles, prior to their first mating, away from their natal area (Greenwood, 1980). Natal dispersal is typically restricted to one sex within a particular species, for example, male red‐backed salamanders (*Plethedon cinereus*) (Liebgold, Brodie, & Cabe, 2011), and female Siberian flying squirrels (Hanski & Selonen, 2009); however, an equal proportion of male and female European roe deer (*Capreolus capreolus*) nataly disperse (Gaillard et al., 2008). For some long‐lived species, adults of either sex may make permanent or long‐term movements to different populations, habitat patches, or herds, for example, desert bighorn sheep (*Ovis canadensis nelsoni*) (Epps, Wehausen, Bleich, Torres, & Brashares, 2007), bison (*Bison bison*) (Plumb, White, Coughenour, & Wallen, 2009), and mountain caribou (*Rangifer tarandus caribou*) (van Oort, McLellan, & Serrouya, 2011). Adult dispersal like natal dispersal is typically sexually biased, with female‐biased dispersal more common in bird species, and male‐biased dispersal more common in mammals (Clobert et al., 2001; Greenwood, 1980). Both natal and adult dispersal are considered important in management and conservation of wild populations as they allow for recolonization of empty habitat patches (Abbott, 2011; Epps, Wehausen, Palsbøll, & McCullough, 2010), demographic rescue (Forester, 2011; van Oort et al., 2011), and maintenance of genetic diversity through gene flow (Bowler & Benton, 2005; Clark, Brown, Stechert, & Zamudio, 2010; Epps et al., 2005).

Adult dispersal is poorly understood compared to natal dispersal, but in some cases may vary widely at the individual level compared to species with strong natal dispersal behaviors. Factors hypothesized to drive adult dispersal include density dependence, often manifesting as a lack of food, interspecific interactions, relatedness of potential mates in the current home range (Bowler & Benton, 2005), or potential for improving social status (Clutton‐Brock & Lukas, 2012). In moose, high population density and resource limitation are correlated with higher adult dispersal rates (Labonté, Ouellet, Courtois, & Bélisle, 1998). Similarly, dispersal of bison in Yellowstone National Park is density dependent and more common as bison populations reach carrying capacity (Plumb et al., 2009), a trend also seen in the fast‐growing population of wood bison in northern Canada (Gates & Larter, 1990; Larter, Sinclair, Ellsworth, Nishi, & Gates, 2000). Thus, changes in animal condition may be a trigger for such movements (Bonte & De La Peña, 2009).

Infectious diseases have been hypothesized as a potential influence on dispersal behavior in animals (Boulinier, McCoy, & Sorci, 2001), through direct impacts on animal behavior (Bowler & Benton, 2005) or indirectly by affecting body condition (Armsworth, 2009; Belthoff & Dufty, 1998; Debeffe et al., 2012). For example, body mass of European roe deer is correlated with dispersal likelihood and dispersal distance, with individuals with a body mass less than 14 kg not dispersing and distance dispersed increasing with body mass (Debeffe et al., 2012). In the same population of European roe deer, dispersal propensity decreases with nematode parasite burden, which is associated with a decrease in body mass (Debeffe et al., 2014). Heeb et al. (1999) observed a similar pattern in the great tit (*Parus major*), where male fledglings from nests infested with hen fleas (*Ceratophyllus gallinae)* were likely to disperse shorter distances than fledglings from uninfested nests. However, Brown and Brown (1992) found that higher hematophagous flea (*Ceratophyllus celsus*) and swallow bug (*Oeciacus vicarious*) burdens led to natal dispersal in American cliff swallows (*Petrochelidon pyrrhonota*).

African buffalo offer a unique opportunity to examine the influences of extrinsic and intrinsic factors, including infectious diseases, on adult dispersal. Buffalo are long‐lived, wide‐ranging, and gregarious, with adult dispersal observed in both sexes (Olff, Ritchie, & Prins, 2002). African buffalo have the weakest genetic structure of all African ungulates studied to date (Lorenzen, Arctander, & Siegismund, 2008) and significant differentiation only at the continental scale (Simonsen, Siegismund, & Arctander, 1998), suggesting that buffalo are less philopatric than other African ungulates (Metzger, Sinclair, Hilborn, Hopcraft, & Mduma, 2010). However, as populations are increasingly restricted by loss of habitat, habitat fragmentation, and human‐made barriers, gene flow among some populations may be decreasing (Epps et al., 2013; Heller, Okello, & Siegismund, 2010), and thus, dispersal, gene flow, and connectivity have become conservation priorities for buffalo management (van Hooft, Groen, & Prins, 2003; Simonsen et al., 1998). Moreover, infectious disease and possible interactions with dispersal are particularly important for this species given the potential for disease interactions with livestock and resulting human‐wildlife conflict. Macroparasites, such as bovine tuberculosis and brucellosis may influence individual condition (Caron, Cross, & du Toit, 2003; Gorsich, Ezenwa, Cross, Bengis, & Jolles, 2015), leading to indirect effects on dispersal behavior. Moreover, African buffalo carry diseases that are of economic concern such as bovine tuberculosis (Renwick, White, & Bengis, 2007), brucellosis (Gorsich, Ezenwa, et al., 2015), and foot‐and‐mouth disease (Dion, Van Schalkwyk, & Lambin, 2011). Thus understanding buffalo ranging behavior (Ryan, Knechtel, & Getz, 2006) can help to inform management decision regarding their potential to cause spillover of disease into both wildlife populations (Bastos, Boshoff, Keet, Bengis, & Thompson, 2000), domestic livestock (Michel et al., 2006) and people in the case of zoonotic infections (Michel et al., 2006).

Although male African buffalo could be regarded as natal dispersers, given that observational and genetic studies suggest that almost all male buffalo disperse away from their natal herds (van Hooft et al., 2003; Prins, 1996; Sinclair, 1977), behavior of females differs. Foundational studies described female African buffalo forming discrete herds with limited interherd movements (Prins, 1996; Sinclair, 1977); but more recent observational and genetic work suggests that dispersal by adult female buffalo is not uncommon (Halley, Vandewalle, Mari, & Taolo, 2002; van Hooft et al., 2003). Epps et al. (2013) also recorded partial migration of both female and male African buffalo, which they defined as seasonal movements. Genetic evidence also suggests female dispersal is common, as Simonsen et al. (1998) found little genetic structure in either maternally inherited mitochondrial DNA or microsatellite (nuclear) loci among buffalo populations across 11 localities in eastern and southern Africa. Furthermore, van Hooft et al. (2003) estimated that 5‐20% of female buffalo older than two years of age dispersed per generation (7 years) based on mitochondrial DNA.

Despite abundant evidence that dispersal is a common feature of the adult African buffalo behavioral repertoire, the social and ecological factors underlying decisions to disperse are not well understood in adult male or female buffalo (Halley et al., 2002; Winnie, Cross, & Getz, 2008). Here, we used telemetry and observation to quantify dispersal behaviors for 304 female African buffalo over a 4‐year period. We hypothesized that female dispersal would be influenced by animal traits, environmental conditions, and disease status. We predicted that likelihood of female dispersal would vary with (1) individual condition, age, pregnancy and lactation status, (2) herd identity, (3) season and year, (4) individual macroparasite burdens, including strongyle nematodes, coccidia and schistosomes. Finally, although we had no clear a priori reason to predict that individual infections like microparasites, including *Mycobacterium bovis* (causative agent of bovine tuberculosis) and *Brucella abortus* (causative agent of brucellosis) would influence dispersal, we evaluated those relationships due to the critical implications of dispersal‐disease interactions for wildlife‐livestock relationships.

## 2 MATERIALS AND METHODS

### 2.1 Study area

**Figure 1:**
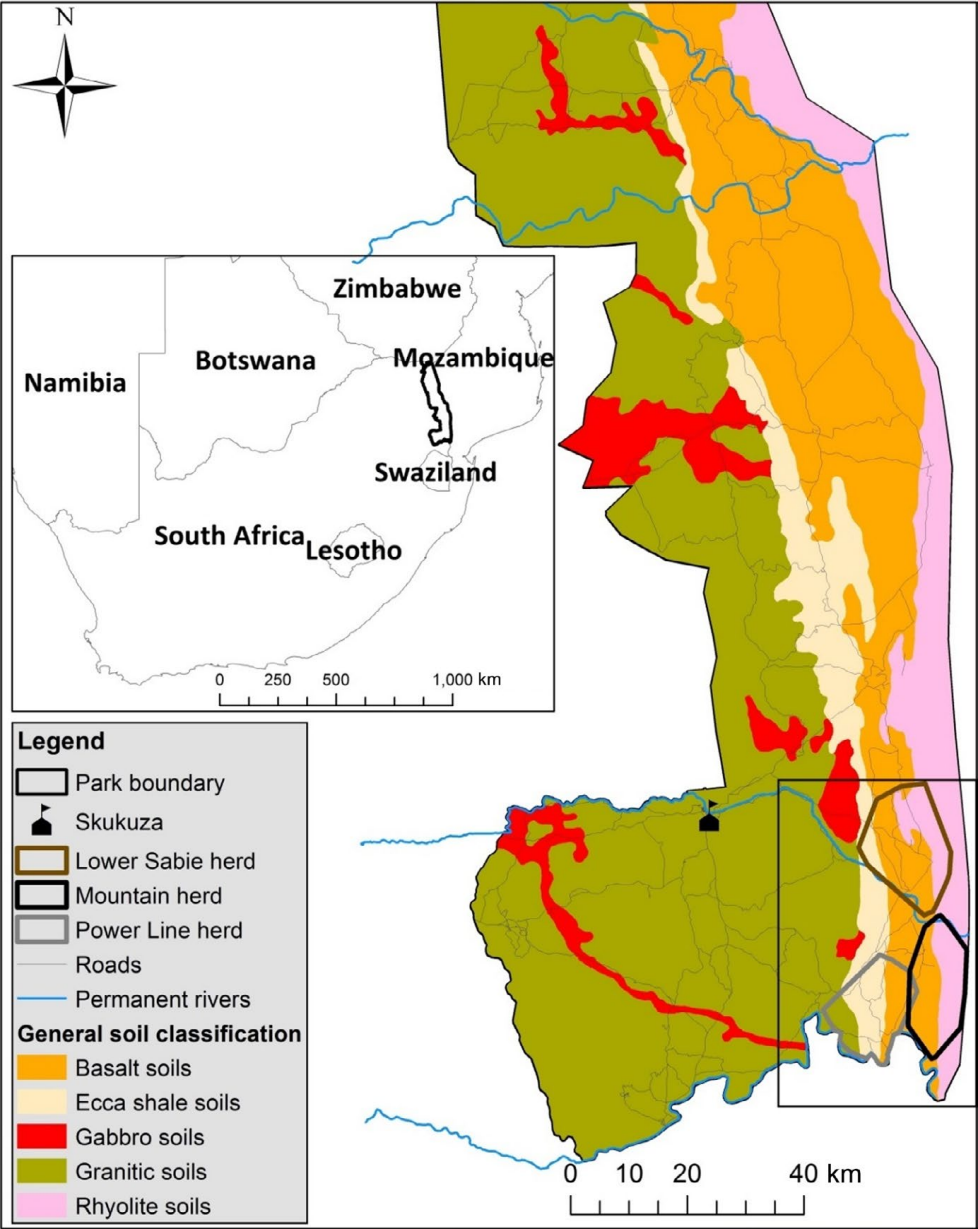
General (black line) and focal study areas (Lower Sabie herd, brown polygon; Mountain herd, black polygon; Power Line herd, gray polygon) for study of dispersal of female African buffalo (*Syncerus caffer*) in Kruger National Park in southern Africa (inset). Initial mass capture of 200 female African buffalo was from the Lower Sabie, Mountain, and Power Line herds in June/July and October of 2008, but females dispersed throughout much of the park during the course of the 4‐year study

Our study took place in the Kruger National Park (KNP), South Africa, which lies between 22.5 and 25.5°S, 31.0 and 31.6°E and is ~19,485 km^2^ in extent (Figure 1). The park has one wet season per year with summer rainfall (October–March) ranging north to south from 400 to 700 mm per year. KNP is located at an average altitude of 250 m (range 200–900 m) above sea level with granitic soils in the west succeeded by Ecca shale, basalt, and rhyolites. The variation in moisture between seasons and soil types, as well as the variation in water holding capacity, gives rise to large regional variations in food availability. The main study area spans the landscape types of the Lebombo mountain bushveld, marula (*Sclerocarya birrea subsp. caffra*)/knobthorn (*Vachellia nigrescens*) savannah, Delagoa thorn (*Vachellia welwitschii*) thickets, Sabie and Crocodile River thickets, and thornveld (Venter, Scholes, & Eckhardt, 2003).

The Lower Sabie (LS) and Crocodile Bridge (CB) herds, although genetically panmictic (Tavalire et al., 2018), utilize distinct areas within KNP with different underlying geological features, which give rise to different soils and associated vegetation (Venter et al., 2003). Water distribution (both natural and artificial) within the areas used by the herds also varies (Gaylard, Owen‐Smith, & Redfern, 2003). This is important since buffalo are water dependent and herd densities are associated with distance to water (Redfern, Grant, Biggs, & Getz, 2003). Opportunities for dispersal also varied for the study herds, due to their position with respect to the park's boundary fence. The fence restricted movement of LS animals to the east, while the CB animals were restricted to both the south and east (Picket, Cadenasso, & Benning, 2003). The CB animals consisted of two herds—Mountain and Power Line herds. We initially considered this a single herd, but after observing the buffalo and inspecting spatial data, we concluded that there were two separate herds. This led to a 4‐way categorical factor with LS_herd, CB_herd1, CB_herd2, and CB_herd1 and 2 combined (CB herd 1 and 2 were combined for the first three out of nine captures).

### 2.2 Capturing buffalo and tracking movement

In June/July and October of 2008, we captured 200 female buffalo, 100 each from two sections in the southern KNP: LS and CB. Age ranges in this dataset at first capture varied from 17 to 156 months of age ($\overset{-}{x}$ = 48 months; *σ* = 23.2) in the LS herd, and from 21 to 168 months ($\overset{-}{x}$ = 54 months; *σ* = 24.6) for the CB herds. Sinclair (1977) classed African buffalo age classes as follows: calves (<1 year), two‐year olds (2–3 years of age), subadults (3–5 years of age), and adults (>5 years of age). Mean age of calving in the Serengeti is 4.8 years of age, although individuals as young as 3.5 years have been recorded calving (Sinclair, 1977). Captures were conducted from helicopters with a dart gun using a combination of etorphine hydrochloride, azaperone, and ketamine. Effects of the opioids on immobilized buffalo were reversed using a combination of naltrexone and diprenorphine. Buffalo were fitted with either a GPS (*n* = 7) or a VHF collar (*n* = 193) as part of a study investigating bovine tuberculosis and gastrointestinal helminth (GI) interactions (Table 2). As part of this study, 100 randomly selected animals (50 from each section) received a long‐acting anthelmintic bolus (Panacur bolus (Intervet, UK)).

We recaptured all buffalo biannually. The timing of the initial captures allowed us to alternate between the two areas twice per year. All LS animals came from one large buffalo herd, while CB buffalo were distributed among two main herds, the Mountain and Power Line herds (Figure 1). Any animals that died throughout the study period were replaced with similar‐aged animals from the same herd. Captures were conducted using the same immobilization method as the initial captures, except that buffalo were darted from the ground using 4 x 4 vehicles. Satellite collars provided 2 locations daily (06:00 and 18:00 local time) to establish the general location of each buffalo herd. Once a herd was located, we verified presence/absence of collared individuals and recorded date, time, and GPS location. All VHF collar frequencies for the entire study area were checked in each herd to detect individuals that dispersed between herds.

Buffalo in the LS herd were monitored throughout the duration of the project. However, with the CB herds (Figure 1) this was not always possible due to a greater distance from our base location at Skukuza and terrain that was difficult to navigate during the wet season. Thus, sample distribution for collar locations in the LS herd was more even throughout the year than sample distribution for the CB herds (Supporting information Figure S2).

Eleven collared buffalo left the confines of KNP; when possible, those individuals were recaptured, brought back into KNP, and placed in the herd closest to the point of exit. When individuals left KNP to unfenced adjoining private reserves, relocation was not possible, so collars were removed. If buffalo crossed the fence on KNP's eastern boundary into the part of Mozambique, collars could not be retrieved.

The LS herd contained satellite collars throughout the entire duration of the project (*n* = 4, with 1 replaced in November 2009 due to predation), but lost 2 to dispersal to different herds during the course of the study. The CB herd had 3 satellite collars throughout the project (Mountain herd, *n* = 2; Power Line herd, *n* = 1).

### 2.3 Definition of dispersal and analysis of spatial data

For the purpose of this study, we defined dispersers as “individual buffalo that were recorded outside of their herd of origin's home range and were joined with another herd, not returning to their herd of origin for at least one season.” A limitation of this study is that individual buffalo were not monitored from conception, and therefore, a distinction between natal and adult dispersal cannot be made. For each herd, GPS locations from the satellite collars were mapped in ArcMap 10.1 (Environmental Systems Research Institute, Inc., Redlands, CA) and home ranges estimated for each of the original herds using a minimum convex polygon (Worton, 1987) in Hawth's Tools (Beyer, 2004). This analysis assumed that buffalo with satellite collars accurately reflected the position of the entire herd. Home ranges were estimated separately for the wet season (October to March) and the dry season (April to September) in each year from September 2009 to August 2012.

### 2.4 Sample collection

Age, condition, pregnancy, and lactation status were recorded for all recaptured buffalo, and the experimental individuals received the Panacur bolus. Blood was collected via jugular venipuncture into 10‐ml EDTA‐coated vacutainer tubes, placed on ice, and transported to Veterinary Wildlife Services (VWS) laboratory in Skukuza, KNP. Feces were collected rectally and transported back to the laboratory in a cooler with ice packs. Age was determined by tooth eruption up to 5 years and then judged by tooth wear (Grimsdell, 1973; Jolles, 2007). Body condition was determined by palpation of fat reserves on the ribs, spine, hips, and tail on a scale of 1 (very poor) to 5 (very good) and then averaged (Ezenwa, Jolles, & O'Brien, 2009). Pregnancy status was determined by rectal palpation (Beechler, Broughton, Bell, Ezenwa, & Jolles, 2012). Lactation was determined by manually milking all four teats (Jolles, Cooper, & Levin, 2005).

### 2.5 Diagnostics

Methods we employed for macro‐ and microparasite diagnostics have been described previously (Budischak, Jolles, & Ezenwa, 2012; Ezenwa, 2003; Gorsich, Ezenwa, et al., 2015). Fecal egg and oocyst counts were used to assess GI nematode and coccidian infection using a modified McMaster method (Ezenwa, 2003). Previous work has shown GI nematode infections in this buffalo population commonly include, *Cooperia fuelleborni*, *Haemonchus placei*, *Haemonchus bedfordi*, and an undescribed *Parabronema* species and that fecal egg counts accurately reflect GI helminth burdens (Budischak, Hoberg, Abrams, Jolles, & Ezenwa, 2015). A lateral flow assay to detect a specific schistosome antigen, circulating anodic antigen (CAA) in serum (Corstjens et al., 2008), was validated for use in buffalo (Beechler et al., 2017) and used to determine schistosome infection. TB was diagnosed using a whole‐blood gamma interferon assay (BOVIGAM, Prionics), optimized for African buffalo with a sensitivity of 86% and a specificity of 92% (Michel, Cooper, Jooste, Klerk, & Jolles, 2011). A commercial enzyme‐linked immunosorbent assay (IDEXX Brucellosis Serum Ab Test) was used to detect brucellosis (Gorsich, Ezenwa, et al., 2015).

### 2.6 Environmental condition

We did not have metrics for environmental condition per se, but season and year reflect variation in body condition associated with variation in rainfall. As evidence of this, Ryan et al. (2012) show that body condition is correlated with fecal nitrogen at a three‐month time lag, and at a one‐month time lag with normalized differential vegetation index (NDVI), which is an index of greenness of the landscape.

### 2.7 Dispersal rates

In order to compare dispersal rates across different African buffalo dispersal studies (Halley et al., 2002; van Hooft et al., 2003), we used a crude measure of number of individuals dispersing per year from the LS and CB. During the course of the four‐year study, 304 female African buffalo were captured and tracked—137 in the LS herd and 167 in the CB herds. Throughout the study period, 93 individuals (30.6%) dispersed, 54 from the LS herd and 39 from the CB area. Thus, 39.4% of LS animals and 23.4% of CB animals dispersed throughout the study period.

Owing to mortalities and dispersal, not all animals were in the study, or in their herd of origin, for the full study period of four years. Annual dispersal rates for each herd were therefore calculated as the number of dispersers observed in each herd, divided by the number of “buffalo‐years‐at‐risk” of dispersal from that herd. The number of buffalo‐years‐at‐risk of dispersal was calculated by scoring, for each buffalo, the duration from its first capture until it either died or left the herd. These individual observation periods were then added up for all buffalo observed in the herd, to give the total number of buffalo‐years‐at‐risk of dispersing. Dispersal rates thus expressed the annual per‐capita “risk” or likelihood of dispersing from a given herd, analogous to incidence rates in epidemiological studies.

### 2.8 Drivers of dispersal

We used generalized linear mixed models (GLMMs) with a binomial distribution (dispersed vs. not dispersed) to explore which variables best predicted whether female buffalo dispersed. Dispersal data refer to the single data point for each individual disperser at the capture and sampling point immediately prior to dispersal. Prior to that point, the data from each disperser contribute to data for its herd of origin (Supporting information Figure S3). Once dispersed, a disperser's data were no longer included in analyses. We conducted two analyses using: (a) all data combined (hereafter, pooled data), (b) the LS herd, and (c) the herds in the CB area. We analyzed herds separately, as drivers of dispersal may differ between populations experiencing different environments. Due to missing data on certain predictor variables, we ran all three models with reduced datasets (Table 1). We then ran the same three models with a reduced set of predictor variables to take advantage of a larger sample of both total observations and dispersal events (Table 1).

**Table 1:** Breakdown of data used in study to determine drivers of dispersal in female African buffalo (*Syncerus caffer*) dispersal

Analyses	Data set	# Fixed effects	# Observations	# Dispersers	Random/nested random effect
Initial	Pooled	13	923	59	Animal ID
Initial	LS herd	12	415	30	Animal ID
Initial	CB herd	12	508	29	Animal ID/Herd ID
Secondary	Pooled	7	1349	91	Animal ID
Secondary	LS herd	6	646	52	Animal ID
Secondary	CB herd	6	703	39	Animal ID/Herd ID

For the initial data set predictor fixed effects included, animal traits, season, year, treatment, GI parasites, and microparasite infections as fixed effects (Table 2) with herd ID included in the pooled data. We generated correlation matrices using raw Pearson correlations between all sets of variables (Hosmer & Lemshow, 2000) (Supporting information Tables S4 and S7). To account for individual variation due to repeated measures. Animal ID was included as a random effect in the pooled and LS herd data. For analyses of the CB data, we used the nested random effects of animal ID and herd identity (Bates, Maechler, Bolker, & Walker, 2015) to account for the repeated measures of individuals within the multiple herds that comprise the CB population. We then reran the same models on a larger data set with fewer predictors, excluding treatment, GI parasites, and microparasite infections. Reproductive measures were kept in the secondary analysis as they did not affect sample sizes.

**Table 2:** Data collected over the course of this study

Measure	Category	Measure type	Statistical measure	Biological significance
Age	Animal trait	Quantitative. Measured in months.	Continuous	Estimated by tooth eruption and wear
Body condition	Animal trait	Condition values. Scored on a 1–5	Continuous	Fat reserves as a measure of body condition^d^
Pregnancy status	Animal trait	Yes/No	Binary	Determined by rectal palpation^a^
Lactation status	Animal trait	Yes/No	Binary	Manual milking of all four teats^g^
Season	Environmental variable	Divided into early wet, late wet, early dry, and late dry	Categorical	Quality and abundance of grazing varies by season^i^
Bolus	Experimental/Treatment	Yes/No	Binary	Reduces nematode burden^e^
Strongyle infection	Gastrointestinal parasites	Quantitative—eggs per gram (logged)	Continuous	Quantifies strongyle infection by FEC^e^
Coccidia infection	Gastrointestinal parasites	Quantitative—oocysts per gram (logged)	Continuous	Quantifies coccidian species infection in feces by quantifying oocysts^b^
Schistosome infection	Gastrointestinal parasites	Continuous—titer (logged)	Continuous	CAA titer used as a proxy for adult schistosome burden^c^
bTB status	Bacteria	Positive/negative for *Mycobacterium bovis*	Binary	Use ELISA to diagnose bTB infection^h^
Brucellosis status	Bacteria	Positive/negative for *Brucella abortus*	Binary	Commercial enzyme‐linked Immunosorbent assay^f^

a Beechler et al. (2012).

b Budischak et al. (2012).

c Corstjens et al. (2008).

d Ezenwa et al. (2009).

e Ezenwa, Etienne, Luikart, Beja‐Pereira, and Jolles (2010).

f Gorsich, Bengis, Bengis, Ezenwa, and Jolles (2015), Gorsich, Ezenwa, et al. (2015).

Grimsdell (1973).

g Jolles et al. (2005).

Jolles (2007).

h Michel et al. (2011).

i Venter et al. (2003).

Using the global model, we generated a complete model set implemented with the dredge function in the *MuMIn* package (Bartoń, 2017), including the null model. We ranked models according to Akaike's Information Criteria corrected for small sample size (AIC*_c_*, Burnham & Anderson, 2002), reporting all models within 2 AIC*_c_* (Supporting information Tables S1 and S5). To explore the importance of each individual predictor, we summed the AIC*_c_* weights for each predictor across all models within 2 AIC*_c_* to calculate each predictor's relative importance (Table 3) (Burnham & Anderson, 2002). We further evaluated the strength of specific effects based on the degree to which 95% confidence intervals for parameter estimates overlapped zero (Grueber, Nakagawa, Laws, & Jamieson, 2011). All statistical analyses were performed in the program R, v. 3.4.2 (R Development Core Team, 2017), with models fitted using the glmer and dredge functions in the *lme4* (Bates et al., 2015) and *MuMIn* (Bartoń, 2017) packages.

**Table 3:** Cumulative AIC*_c_* weights (Ʃ *w_i_*) or relative importance values from initial analyses for each fixed effect from generalized linear mixed effects models predicting dispersal likelihood of female African buffalo (*Syncerus caffer*) in Kruger National Park, South Africa, for the period June 2008–August 2012. Maximum cumulative weight (Ʃ *w*_i_) or relative importance for each predictor variable is 1; explanatory variables varied by data set but included condition, age, pregnancy status, lactation status, season, year, strongyle and coccidia burden, schisto and bovine tuberculosis and brucellosis infection status. Bolded model parameters were included in the secondary analysis

	Ʃ *w_i_*		
Model parameter	Pooled	Lower Sabie	Crocodile Bridge
**Condition**	1.00	0.19	0.84
**Age**	1.00	1.00	0.04
**Pregnant**	0.03	0.19	0.05
**Lactation**	0.59	0.28	0.04
**Season**	0.00	1.00	0.00
**Year**	1.00	1.00	0.09
**Herd**	0.68	NA	NA
Treatment	0.03	0.08	0.09
Strongyle	0.12	0.00	0.05
Coccidia	0.00	0.00	0.04
Schisto	0.49	0.00	0.77
bTB	0.31	0.11	0.15
Brucella	0.22	0.08	0.22

### 2.9 Ethics statement

All procedures were approved by South African National Parks Board (Reference No. SPARS914) and by Oregon State University and University of Georgia IACUCs (Protocol numbers: OSU No. 2833, UGA No. A201010‐190‐A1).

## 3 RESULTS

### 3.1 Dispersal rates

The annual dispersal rate for the LS herd was higher at 19.2% than the annual dispersal rate of 14.5% in the CB area. This is much higher than the crude estimate one would arrive at by simply dividing the fraction of animals that dispersed (here, 30.6%) by the study duration of four years, that is, 7.6% overall, or 9.9% and 5.9% for the LS and CB herds, respectively. These crude estimates would be misleading because they erroneously assume that all buffalo, including dispersers and nondispersers, were observed in their herds of origin. Mean dispersal distance as determined by distance dispersed from the last capture location for the LS herd was 35.80 km (range: 2.23‐110.19 km, n = 52), while the mean dispersal distance from the CB herds was 25.68 km (range: 0.55‐98.81 km, n = 39) (Supporting information Figure S4). Times between pre‐dispersal capture points and the post‐dispersal location points varied due to the difficulty in locating some of the dispersers post‐dispersal.

### 3.2 Drivers of dispersal

A number of the infectious disease variables used to predict probability of dispersal in the initial analyses occurred in the top 2 AIC*_c_* of models, including GI parasites (strongyles, coccidia, and schistosomes) and microparasite infections (bovine tuberculosis)  (Supporting information Table S1). However, none of the disease variables had a high relative importance factor, and the 95% CIs for the parameter estimates overlapped zero, that is, limiting the importance of disease covariates (Supporting information Table S2). Treatment (antihelminthic bolus) had no effect on dispersal (Supporting information Table S1).

**Figure 2:**
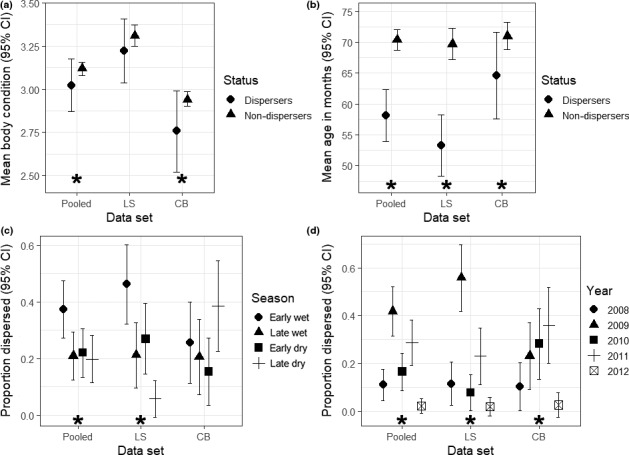
Predictors of female African buffalo (*Syncerus caffer*) dispersal likelihood in the pooled, Lower Sabie, and Crocodile Bridge data, Kruger National Park for the period, June 2008–August 2012, generated using the means raw values from the secondary analysis (pooled ‐ dispersers (n = 91), non‐dispersers (n = 1,258); LS ‐ dispersers (n = 52), non‐dispersers (n = 594); CB ‐ dispersers (n=39), non‐dispersers (n = 664)) for (a) comparison of mean condition, (b) comparison of mean age in months, (c) proportion dispersed by season, and (d) proportion dispersed by year. ^*^indicates whether the explanatory variable had an importance value greater than 0.90, and whether or not the 95% confidence intervals for parameter estimates in the GLMMs overlapped zero

For the larger data set with the reduced set of variables, we used to predict the effects of animal traits, reproductive status, environmental variables, on probability of dispersal we found that across study regions, body condition, age, herd identity, season, and year contributed to variation in dispersal (Table 4). However, the relative importance of these drivers of dispersal differed between the two study areas, LS and CB (Table 4). The dispersal rate for LS was higher than for CB herds. Younger buffalo were more likely to disperse (Table 4, Figure 2b). This trend was driven by a strong, negative effect of age on dispersal rate in the LS and CB herds.

**Table 4:** Summary results of secondary analyses for each fixed effect from generalized linear mixed effects models after model averaging: predicting dispersal likelihood of female African buffalo (*Syncerus caffer*) in Kruger National Park, South Africa, for the period June 2008–August 2012. Maximum cumulative weight (Ʃ *w*_i_) or relative importance for each predictor variable is 1; explanatory variables varied by data set but included condition, age, pregnancy status, lactation status, season, and year.

Data set	Parameter	Effect on likelihood of dispersal	Odds ratio	Odds ratio (95% CI)	Estimate	Unconditional *SE*	Ʃ *w_i_*
Pooled	Intercept				−0.75	0.81	–
	Condition	**↓**			−0.57	0.19	1.00
	Age	**↓**			−0.02	0.01	1.00
	Pregnant	↓	1.24	(0.71; 2.16)	−0.22	0.19	0.33
	Lactation	↓	1.88	(0.80; 4.40)	−0.63	0.45	0.54
	Herd_Lower Sabie	**↑**	3.96	(1.90; 8.22)	1.37	0.37	1.00
	Herd_Mountain	**↑**	3.64	(1.15; 11.51)	1.29	0.59	1.00
	Herd_Power Line	**↑**	5.40	(1.99; 14.71)	1.69	0.51	1.00
	Year_2009	**↑**	2.23	(1.04; 4.78)	0.80	0.39	1.00
	Year_2010	↓	1.12	(0.40; 3.10)	−0.11	0.52	1.00
	Year_2011	↑	2.13	(0.79; 5.76)	0.76	0.51	1.00
	Year_2012	↓	2.86	(0.52; 15.82)	−1.05	0.87	1.00
Lower Sabie	Intercept				−0.63	0.71	–
	Age	**↓**			−0.03	0.01	1.00
	Pregnant	↓	1.94	(0.86; 4.36)	−0.66	0.46	0.62
	Lactation	↓	2.42	(0.61; 9.61)	−0.88	0.66	0.48
	Season_late wet	**↓**	3.99	(1.64; 9.73)	−1.38	0.45	1.00
	Season_early dry	**↓**	2.52	(1.12; 5.68)	−0.92	0.41	1.00
	Season_late dry	**↓**	9.56	(2.51; 36.43)	−2.26	0.68	1.00
	Year_2009	**↑**	4.55	(1.57; 13.15)	1.51	0.54	1.00
	Year_2010	↑	1.82	(0.43; 7.73)	0.60	0.74	1.00
	Year_2011	**↑**	7.18	(1.87; 27.60)	1.97	0.69	1.00
	Year_2012	↑	4.41	(0.35; 55.61)	1.49	1.29	1.00
Crocodile Bridge	Intercept				−0.51	1.09	–
	Condition	**↓**			−0.65	0.29	1.00
	Age	**↓**			−0.02	0.01	1.00
	Pregnant	↑	1.24	(0.58; 2.64)	0.22	0.21	0.23
	Lactation	↓	1.18	(0.38; 3.64)	−0.17	0.27	0.21
	Year_2009	↑	1.47	(0.43; 5.01)	0.38	0.63	1.00
	Year_2010	↑	3.05	(0.86; 10.77)	1.12	0.64	1.00
	Year_2011	**↑**	5.28	(1.43; 19.54)	1.66	0.67	1.00
	Year_2012	↓	1.71	(0.16; 17.91)	−0.54	1.20	1.00

Bolded symbols indicate parameters where the odds ratio (95% CI) do not overlap 1.

Dispersal likelihood varied interannually (Table 4, Figure 2d), but different years had high dispersal rates in two areas. Because our observation period started in July 2008 and ended in June 2012, we only had three entire years (2009–2011) among which to compare dispersal rates. The driest year, 2009, stands out as having very high dispersal rates, driven by strong movement out of the LS herd (Table 4, Figure 2d). Overall, most adult buffalo dispersal occurred in the early wet season in the LS herd, when buffalo are in their worst condition, following resource restriction throughout the dry season. Interestingly, the LS herd drove this effect, while dispersal from the CB herds peaked earlier, in the late dry season (Table 4, Figure 2c). This corresponds to an earlier loss of body condition for buffalo in the CB area, likely due to differences in rainfall patterns and soil type between the two areas. Mean late dry season (July–September) rainfall for the LS area was 101.9 mm vs. 89.1 mm for the CB area (Supporting information Figure S5).

Dispersing buffalo tended to be in poorer body condition than comparable individuals that did not leave their herd. This trend was stronger in the CB herds (Table 4, Figure 2a), where body condition was overall much worse than at LS (Gorsich, Ezenwa, et al., 2015). Finally, there was no evidence that reproductive status affected dispersal, as relative importance values were low, with confidence intervals for reproductive parameters (pregnancy and lactating status) consistently overlapping zero (Table 4).

## 4 DISCUSSION

Adult dispersal was common in female African buffalo and appeared to be driven by resource restriction. Buffalo dispersed after the dry season, following prolonged resource restriction, and individuals in poor body condition were more likely to disperse. By contrast, none of the infectious diseases we diagnosed directly affected dispersal decisions in female adult buffalo. Buffalo thus appear to disperse as a mechanism for escaping resource limitation, which could occur due to external factors such as drought resulting in intraspecific competition in areas where buffalo populations are approaching seasonal carrying capacities. Matthysen (2012) suggests that increased population density can modify habitat quality, influencing dispersal propensity, for example, western bluebirds (*Sialia mexicana*) produce more aggressive young with high dispersal propensity when nest cavities are limited (Potticary & Duckworth, 2018). Kapota et al. (2016) in an experiment, restricting food sources, found an increased dispersal propensity in golden jackals (*Canis aureus*); however, under the same conditions red foxes (*Vulpes vulpes*) did not show an increased dispersal propensity. *Erigone arctica* and *E. dentiplapis* when starved also showed a decreased propensity to disperse (Bonte, Lukáč, & Lens, 2008), while increased density dependence did not impact female red deer (*Cervus elaphus*), but decreased male dispersal propensity and increased dispersal distance (Loe, Mysterud, Veiberg, & Langvatn, 2009).

We observed slightly differing patterns in the study herds. In the LS herd, young buffalo were more likely to disperse, whereas both young buffalo and buffalo in poor condition were more likely to disperse in the CB herds. This pattern matches predictions from theoretical explorations of dispersal rates. For instance, Bonte and De La Peña (2009) argued that dispersal rates would increase for individuals with higher body condition when there is connectivity across metapopulations as well as environmental variation and that individuals in good body condition may disperse to take advantage of low‐density areas that could offer increased breeding opportunities. Young buffalo in the LS herd were in relatively good condition but may have faced strong intraspecific competition for resources due to the hierarchical social structure of buffalo herds (Mloszewski, 1983; Sinclair, 1977). Bonte and De La Peña (2009) predict that individuals in low body condition will disperse more frequently when mortality is high, and there is limited variation. Ryan, Knechtel, and Getz (2007) theorized that a lack of resources associated with low rainfall years would lead to individual female buffalo becoming nonreproductive or dispersing to better habitat. This alternate pattern may explain why individuals in poor condition in the more resource‐limited CB herd were willing to risk dispersal; that is, both herds experience condition‐dependent dispersal (Ims & Hjermann, 2001). However, the underlying mechanisms differ.

Animal movements such as migration and dispersal offer individuals the opportunity to flee from infected habitats and thereby avoid highly infected individuals not able to migrate or disperse (Altizer, Bartel, & Han, 2011; Hall, Altizer, & Bartel, 2014). Conversely, it has been suggested that stressful movements, such as dispersal and migration, could potentially lower immunological function, making individuals more susceptible to infection (Bonte et al., 2012). We found no direct effect of infectious diseases on the likelihood of dispersal in our study. However, the relationship we found between dispersal and condition, and the fact that schistosome burden in the data set with the full set of predictors contributed to the top model without having a direct effect, points to the possibility that some infectious diseases, especially chronic infections, could indirectly impact dispersal if they negatively affect body condition, for example, bovine tuberculosis (Caron et al., 2003) and GI parasites (Beechler et al., 2017; Budischak et al., 2012).

Here, we have reported one of the most extensive studies on dispersal in a large mammal species, recording 550 buffalo years and 93 dispersal events over a four‐year observation period. Although there has been a number of studies showing a link between resource limitation and dispersal large ungulates (Gaillard et al., 2008; Loe et al., 2009), our study revealed, for the first time, a tight link between resource limitation and dispersal decisions in a subtropical, social ungulate. Adult buffalo appear to disperse in an attempt to outrun unfavourable resource conditions, which can cause loss of body condition and potentially, fitness. In the context of subtropical climates, where season and interannual variation in forage availability are largely driven by rainfall patterns, our results suggest that dispersal patterns for buffalo and possibly other large herbivores may change fundamentally due to anthropogenic climate change and surface water management. Most (80%) climate projections suggest that southern Africa will have increased temperatures, reduced rainfall, and later onset of rains (James & Washington, 2013). Our study suggests that such changes would increase adult dispersal rates in African buffalo, with likely consequences for disease spread. As such, future research should address effects of dispersal on fitness‐related variables such as lifetime reproductive potential, survival, genetic diversity of offspring, and resistance to infection.

## CONFLICT OF INTEREST

None declared.

## AUTHOR'S CONTRIBUTIONS

All authors conceived the ideas and designed the methodology; RSS, VOE, and AEJ collected the data; RSS led the analysis and writing of the manuscript. All authors contributed critically to the drafts and gave final approval for publication.

## Data Availability

Data are available from Dryad digital repository (https://doi.org/10.5061/dryad.gm27hj3).
