# Supplementary material for: Why did the buffalo cross the park? Resource shortages, but not infections, drive dispersal in female African buffalo (Syncerus caffer)
Source: Ecol Evol. 2019 Apr 30;9(10):5651–63. doi: 10.1002/ece3.5145 (PMC6540691; doi:10.1002/ece3.5145)
Supplement: Supplementary file 1 [file ECE3-9-5651-s001.docx]

**Supporting Information**


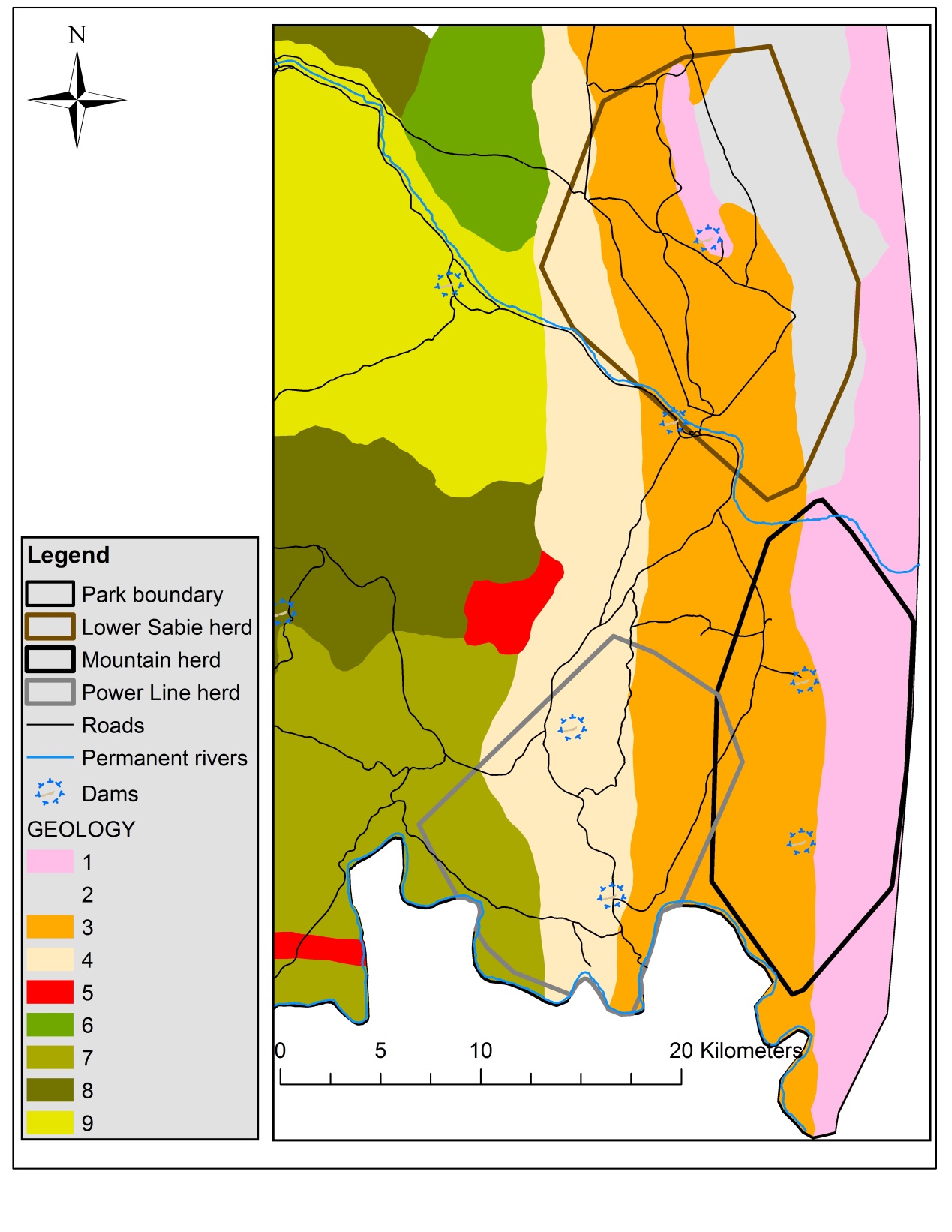


**Fig. S1.** Box area from figure 1 additionally showing locations of artificial water sources and detailed soil derived geology: 1 rhyolite/dactite soils; 2 rhyolite/basalt soils; 3 basalt soils; 4 ecca shale/mudstone soils; 5 Timbivati gabbro soils and 6-9 various granite/gneiss soils.

**
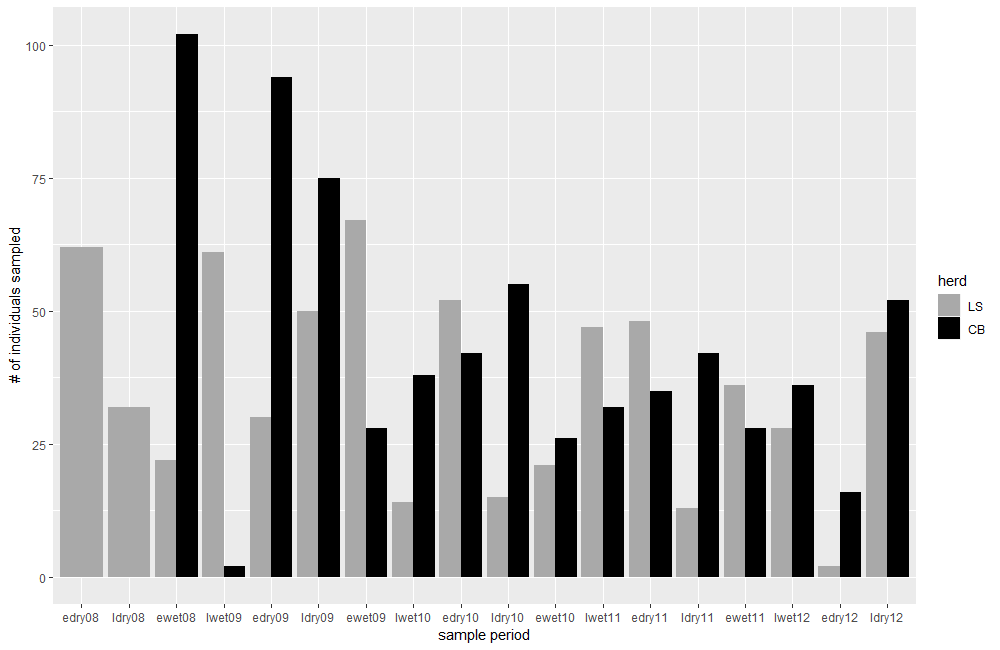
**

**Fig. S2.** Sample distribution showing the number of individuals that were included in the analyses, captured and sampled by season (early dry; late dry; early wet; late wet), and year (2008-2012), across the two study herds (LS – Lower Sabie; CB – Crocodile Bridge). The number of individuals included in the analyses decreased over time, as dispersed individual’s data was excluded from analyses post-dispersal. Season of dispersal was determined by last point observed with herd of origin, not season of capture.

**
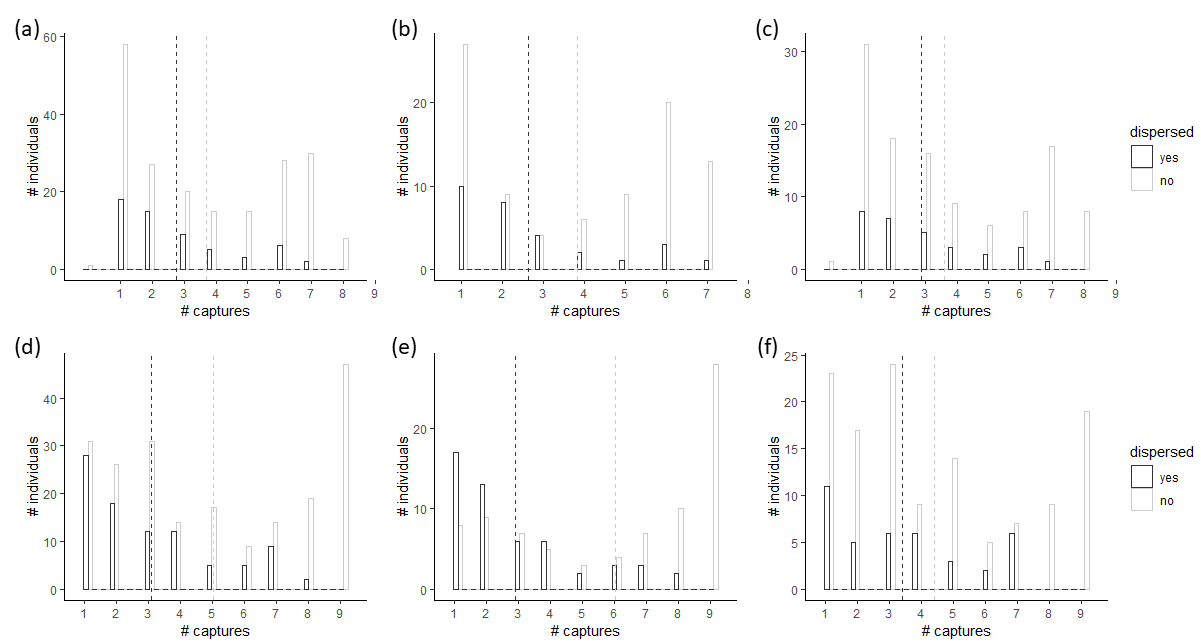
**

**Fig. S3.** Histograms displaying the number of capture events on the x-axis, with the y-axis indicating the number of individuals caught by capture. The black bars indicates dispersers, whose number of captures were counted up until, and including the capture prior to departure. The grey bars indicate the total number of times each non-disperser were captured. The mean number of captures, displayed with dash lines, for dispersers and non-dispersers in the initial analysis was: (a) 2.76 and 3.71 – pooled data; (b) 2.62 and 3.83 – LS data; (c) 2.90 and 3.61 – CB data; and in the secondary analysis was: (d) 3.10 and 5.02 – pooled data; (e) 2.88 and 6.01 – LS data; (f) 3.38 and 4.39 – CB data. The mean number of captures includes the data from the capture point of dispersers immediately prior to dispersal.


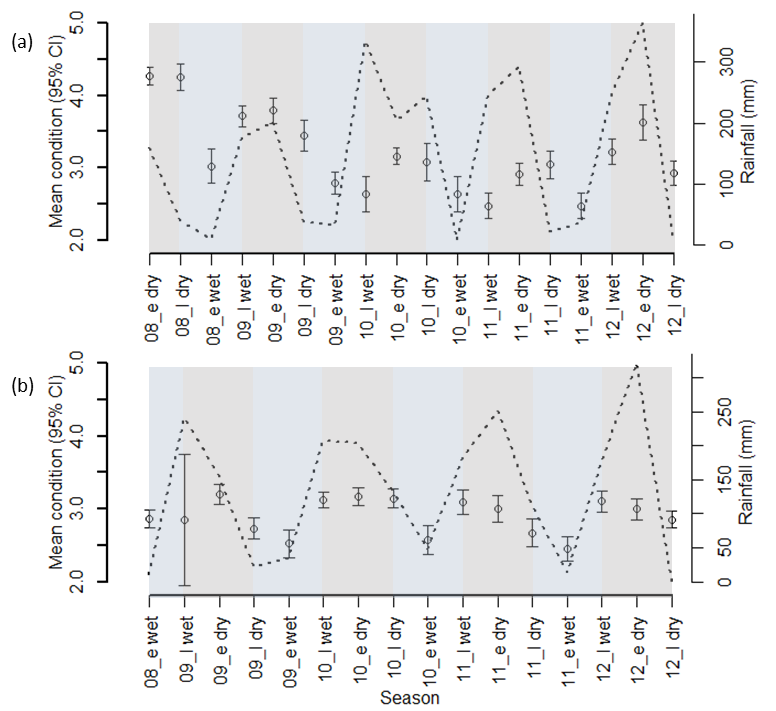


**Fig. S4.** Mean body condition for African buffalo (*Syncerus caffer*) in the (a) Lower Sabie herd and (b) Crocodile Bridge herds, showing a single season (3-month) time lag in variation in mean body condition with 95% confidence intervals, by season (x-axis) and seasonal rainfall (mm) (dash line) for the period, June 2008 to August 2012. Body condition for was assessed at each sampling occasion (for a breakdown of the sample distribution – see Fig. S2). Ryan et al. (2012) found that body condition lags normalized differential vegetation index (NDVI) by 1-month, and fecal nitrogen by 3-months.

**Table S1.** Breakdown of data used in study to determine drivers of dispersal in female African buffalo (*Syncerus caffer*) dispersal.

| Analyses | Data set | # Fixed effects | # Observations | # Dispersers | Random/Nested random effect |
| --- | --- | --- | --- | --- | --- |
| Initial | Pooled | 13 | 923 | 59 | Animal ID |
| Initial | LS herd | 12 | 415 | 30 | Animal ID |
| Initial | CB herd | 12 | 508 | 29 | Animal ID/Herd ID |
| Secondary | Pooled | 7 | 1349 | 91 | Animal ID |
| Secondary | LS herd | 6 | 646 | 52 | Animal ID |
| Secondary | CB herd | 6 | 703 | 39 | Animal ID/Herd ID |

**Table S2.** Model selection of generalized linear mixed effect models for the initial analyses predicting dispersal likelihood of female African buffalo (*Syncerus caffer*) in Kruger National Park, South Africa for the period June 2008 – August 2012, based on Akaike’s Information Criterion (AIC*_c_*) corrected for small sample size. We present all models within 2 AIC*_c_* for the pooled data (923 observations total; 59 dispersal observations), Lower Sabie data (415 observations total; 30 dispersal observations) and Crocodile Bridge data (508 observations total; 29 dispersal observations). Model terms included in final models: condition (continuous), age (continuous), pregnancy and lactating status (binary), season (categorical), year (categorical), treatment (binary), strongyle and coccidia burden (continuous), schistosome titer (continuous), bovine tuberculosis and brucella status (binary), and herd (categorical).

| Data set | Models | K | AIC*_c_* | ∆AIC*_c_* | AIC*_c_*Wt |
| --- | --- | --- | --- | --- | --- |
| Pooled | condition+age+lactation+year+herd | 13 | 423.92 | 0.00 | 0.07 |
|  | condition+age+year+herd | 12 | 424.19 | 0.27 | 0.06 |
|  | condition+age+lactation+year+schisto | 11 | 424.27 | 0.35 | 0.06 |
|  | condition+age+lactation+year+bTB+herd | 14 | 424.28 | 0.36 | 0.06 |
|  | condition+age+year+bTB+herd | 13 | 424.43 | 0.51 | 0.05 |
|  | condition+age+lactation+year+schisto+herd | 14 | 424.63 | 0.71 | 0.05 |
|  | condition+age+year+schisto | 10 | 424.75 | 0.82 | 0.05 |
|  | condition+age+lactation+year+schisto+bTB+herd | 15 | 424.95 | 1.03 | 0.04 |
|  | condition+age+lactation+year+brucella+herd | 14 | 424.99 | 1.06 | 0.04 |
|  | condition+age+year+schisto+herd | 13 | 425.02 | 1.10 | 0.04 |
|  | condition+age+lactation+year+schisto | 12 | 425.22 | 1.30 | 0.04 |
|  | condition+age+lactation+year+schisto+brucella | 12 | 425.24 | 1.32 | 0.04 |
|  | condition+age+year+brucella+year | 13 | 425.25 | 1.32 | 0.04 |
|  | condition+age+schisto+bTB+brucella+herd | 14 | 425.26 | 1.33 | 0.04 |
|  | condition+age+lactation+year+strongyle+schisto | 12 | 425.31 | 1.39 | 0.03 |
|  | condition+age+year+strongyles+schisto | 11 | 425.61 | 1.69 | 0.03 |
|  | condition+age+lacatation+year+strongyle+herd | 14 | 425.62 | 1.69 | 0.03 |
|  | condition+age+lacation+year+bTB+brucella+year | 15 | 425.64 | 1.71 | 0.03 |
|  | condition+age+pregnant+lactation+year+herd | 14 | 425.65 | 1.73 | 0.03 |
|  | condition+age+schisto+brucella+year | 11 | 425.65 | 1.73 | 0.03 |
|  | condition+age+lactation+treatment+year+herd | 14 | 425.68 | 1.76 | 0.03 |
|  | condition+age+year+schisto+bTB | 11 | 425.68 | 1.76 | 0.03 |
|  | condition+age+lactation+year | 10 | 425.74 | 1.82 | 0.03 |
|  | condition+age+year+strongyle+herd | 13 | 425.76 | 1.84 | 0.03 |
|  | condition+age+year+bTB+brucella+herd | 14 | 425.79 | 1.87 | 0.03 |
|  | condition+age+lactation+year+schisto+brucella+herd | 15 | 425.81 | 1.89 | 0.03 |
| Lower | age+season+year | 11 | 201.06 | 0.00 | 0.21 |
| Sabie | age+lactation+season | 12 | 202.13 | 1.07 | 0.12 |
|  | condition+age+season+year | 12 | 202.25 | 1.19 | 0.11 |
|  | age+season+year+bTB | 12 | 202.31 | 1.25 | 0.11 |
|  | age+pregnant+season+year | 12 | 202.38 | 1.33 | 0.11 |
|  | age+season+year+brucella | 12 | 202.71 | 1.66 | 0.09 |
|  | age+season+year+treatment | 12 | 202.96 | 1.90 | 0.08 |
|  | age+pregnant+lactation+season+year | 13 | 202.97 | 1.91 | 0.08 |
|  | condition+age+lactation+season+year | 13 | 202.97 | 1.91 | 0.08 |
| Crocodile | condition+schisto | 6 | 227.64 | 0.00 | 0.08 |
| Bridge | condition+schisto+brucella | 7 | 228.06 | 0.42 | 0.06 |
|  | schisto | 5 | 228.12 | 0.47 | 0.06 |
|  | condition | 5 | 228.39 | 0.75 | 0.05 |
|  | (Intercept only) | 4 | 228.41 | 0.77 | 0.05 |
|  | condition+schisto+bTB | 7 | 228.45 | 0.81 | 0.05 |
|  | schisto+brucella | 6 | 228.73 | 1.09 | 0.05 |
|  | condition+brucella | 6 | 228.75 | 1.10 | 0.05 |
|  | condition+age+schisto | 7 | 228.76 | 1.11 | 0.05 |
|  | brucella | 5 | 228.95 | 1.31 | 0.04 |
|  | condition+treatment+schisto | 7 | 229.17 | 1.53 | 0.04 |
|  | condition+age | 6 | 229.27 | 1.63 | 0.03 |
|  | condition+treatment+schisto+brucella | 8 | 229.29 | 1.65 | 0.03 |
|  | condition+treatment+schisto+bTB | 8 | 229.30 | 1.66 | 0.03 |
|  | condition+age+year+schisto | 11 | 229.32 | 1.68 | 0.03 |
|  | condition+bTB | 6 | 229.34 | 1.70 | 0.03 |
|  | condition+lactation+schisto | 7 | 229.35 | 1.71 | 0.03 |
|  | condition+schisto+strongyle | 7 | 229.39 | 1.75 | 0.03 |
|  | schisto+bTB | 6 | 229.42 | 1.77 | 0.03 |
|  | age+schisto | 6 | 229.49 | 1.84 | 0.03 |
|  | condition+pregnant+schisto | 7 | 229.51 | 1.87 | 0.03 |
|  | condition+age+year | 10 | 229.56 | 1.92 | 0.03 |
|  | condition+schisto+coccidia | 7 | 229.62 | 1.98 | 0.03 |
|  | age | 5 | 229.63 | 1.99 | 0.03 |

^1^ Assessed using the residuals of condition on age due to high correlations between condition and age

^2^ Assessed as early wet, late wet, early dry and late dry

^3^ Assessed as 2008, 2009, 2010, 2011, 2012

**Table S3.** Summary results of initial analyses for each fixed effect from generalized linear mixed effects models after model averaging: predicting dispersal likelihood of female African buffalo (*Syncerus caffer*) in Kruger National Park, South Africa for the period June 2008 – August 2012. Maximum cumulative weight (Ʃ *w_i_*) or relative importance for each predictor variable is 1; explanatory variables varied by data set but included condition, age, pregnancy status, lactation status, season, year, strongyle and coccidia burden, schisto and bovine tuberculosis and brucellosis infection status.

| Data set | Parameter | Effect on likelihood of dispersal | Odds ratio | Odds ratio (95% CI) | Estimate | Unconditional SE | Ʃ *w_i_* |
| --- | --- | --- | --- | --- | --- | --- | --- |
| Pooled | intercept |  |  |  | -0.81 | 1.10 | - |
|  | **condition** | **↓** | **1.78** | **(1.12; 2.84)** | **-0.58** | **0.24** | **1.00** |
|  | **age** | **↓** | **1.02** | **(1.01; 1.03)** | **-0.02** | **0.01** | **1.00** |
|  | pregnant | ↓ | 1.20 | (0.64; 2.26) | -0.18 | 0.06 | 0.03 |
|  | lactation | ↓ | 2.12 | (0.77; 5.85) | -0.75 | 0.54 | 0.59 |
|  | herd_Lower Sabie | ↑ | 2.97 | (1.19; 7.44) | 1.09 | 0.64 | 0.68 |
|  | herd_Mountain | ↑ | 2.11 | (0.52; 8.56) | 0.75 | 0.68 | “ |
|  | herd_Power Line | ↑ | 3.94 | (1.22; 12.74) | 1.37 | 0.81 | “ |
|  | **year_2009** | **↑** | **3.86** | **(1.22; 12.24)** | **1.35** | **0.59** | **1.00** |
|  | year_2010 | ↑ | 1.65 | (0.40; 6.90) | 0.50 | 0.73 | “ |
|  | **year_2011** | **↑** | **4.48** | **(1.09; 18.44)** | **1.50** | **0.72** | **“** |
|  | year_2012 | ↑ | 1.78 | (0.25; 12.85) | 0.58 | 1.01 | “ |
|  | treatment | ↓ | 1.17 | (0.67; 2.03) | -0.15 | 0.05 | 0.03 |
|  | bTB | ↓ | 1.49 | (0.78; 2.83) | -0.40 | 0.26 | 0.31 |
|  | brucella | ↓ | 1.34 | (0.73; 2.47) | -0.29 | 0.19 | 0.22 |
|  | schisto | ↓ | 1.39 | (0.91; 2.12) | -0.33 | 0.22 | 0.49 |
|  | strongyle | ↓ | 1.12 | (0.85; 1.47) | -0.12 | 0.06 | 0.12 |
| Lower | intercept |  |  |  | -1.34 | 1.33 | - |
| Sabie | condition | ↓ | 1.50 | (0.69; 3.26) | -0.40 | 0.24 | 0.19 |
|  | **age** | **↓** | **1.03** | **(1.01; 1.06)** | **-0.03** | **0.12** | **1.00** |
|  | pregnant | ↓ | 1.57 | (0.64; 3.87) | -0.08 | 0.27 | 0.19 |
|  | lactation | ↓ | 2.34 | (0.47; 11.64) | -0.85 | 0.58 | 0.28 |
|  | season_late wet | ↓ | 1.50 | (0.34; 6.57) | -0.40 | 0.75 | 1.00 |
|  | season_early dry | ↓ | 1.34 | (0.46; 3.96) | -0.30 | 0.55 | “ |
|  | **season_late dry** | **↓** | **13.44** | **(1.60; 112.72)** | **-2.60** | **1.09** | **“** |
|  | **year_2009** | **↑** | **1.50** | **(8.45; 47.67)** | **2.13** | **0.88** | **1.00** |
|  | year_2010 | ↑ | 1.49 | (0.17; 12.71) | 0.40 | 1.09 | “ |
|  | **year_2011** | **↑** | **8.22** | **(1.16; 58.05)** | **2.11** | **0.99** | **“** |
|  | year_2012 | **↑** | 6.99 | (0.31; 155.47) | 1.95 | 1.58 | “ |
|  | treatment | ↓ | 1.20 | (0.54; 2.68) | -0.18 | 0.13 | 0.08 |
|  | bTB | ↓ | 1.48 | (0.63; 3.48) | -0.40 | 0.19 | 0.11 |
|  | brucella | ↓ | 1.33 | (0.57; 3.15) | -0.29 | 0.16 | 0.09 |
| Crocodile | intercept |  |  |  | -1.21 | 1.22 | - |
| Bridge | condition | ↓ | 1.70 | (0.90; 3.23) | -0.53 | 0.33 | 0.70 |
|  | age | ↓ | 1.01 | (0.99; 1.03) | -0.01 | 0.01 | 0.20 |
|  | pregnant | ↑ | 1.19 | (0.53; 2.66) | 0.17 | 0.08 | 0.03 |
|  | lactation | ↓ | 1.43 | (0.41; 5.01) | -0.36 | 0.13 | 0.03 |
|  | year_2009 | ↑ | 2.67 | (0.51; 13.89) | 0.98 | 0.32 | 0.06 |
|  | year_2010 | ↑ | 3.67 | (0.68; 19.83) | 1.30 | 0.39 | “ |
|  | **year_2011** | **↑** | **6.92** | **(1.28; 37.47)** | **1.93** | **0.52** | **“** |
|  | year_2012 | ↑ | 1.86 | (0.14; 25.20) | 0.62 | 0.37 | “ |
|  | treatment | ↑ | 1.37 | (0.33; 1.58) | -0.32 | 0.13 | 0.07 |
|  | schisto | ↓ | 1.55 | (0.91; 2.61) | -0.43 | 0.30 | 0.68 |
|  | strongyle | ↓ | 1.10 | (0.78; 1.57) | -0.09 | 0.04 | 0.03 |
|  | coccidia | ↓ | 1.06 | (0.69; 1.62) | -0.06 | 0.04 | 0.03 |
|  | bTB | ↓ | 1.58 | (0.62; 4.06) | -0.46 | 0.25 | 0.15 |
|  | brucella | ↓ | 1.68 | (0.72; 3.93) | -0.52 | 0.32 | 0.26 |

**Table S4.** Univariate models of fixed effects for the initial analyses predicting dispersal likelihood of female African buffalo (pooled data (923 observations total; 59 dispersal observations), Lower Sabie data (415 observations total; 30 dispersal observations) and Crocodile Bridge data (508 observations total; 29 dispersal observations)). Univariates listed below include condition (continuous), age (continuous), pregnancy and lactating status (binary), herd (categorical), season (categorical), year (categorical), strongyle and coccidia burden (continuous), schistosome titer (continuous), and bovine tuberculosis and brucella status (binary), with the nested random effect of (1|Animal_ID/Herd_ID) or the random effect of Animal_ID to account for pseudo replication and to acknowledge the different herds.

| Data set | Predictor | Effect on likelihood of dispersal | Odds-  ratio | Odds ratio (95% CIs) | Estimate | SE | p-value |
| --- | --- | --- | --- | --- | --- | --- | --- |
| Pooled | condition | ↓ |  |  | -0.42 | 0.20 | 0.037 |
|  | age | ↓ |  |  | -0.01 | 0.01 | 0.032 |
|  | pregnant |  | 0.95 | (0.54; 1.66) | -0.05 | 0.28 | 0.857 |
|  | lactation | (↓) | 2.37 | (0.93; 6.02) | -0.86 | 0.48 | 0.070 |
|  | ^1^season_late wet |  | 0.71 | (0.33; 1.51) | -0.35 | 0.39 | 0.371 |
|  | ^1^season_early dry |  | 0.60 | (0.30; 1.18) | -0.52 | 0.35 | 0.138 |
|  | ^1^season_late dry | (↓) | 2.05 | (0.97; 4.36) | -0.72 | 0.38 | 0.061 |
|  | ^2^year_2009 | ↑ | 4.17 | (1.40; 12.39) | 1.43 | 0.56 | 0.010 |
|  | ^2^year_2010 |  | 2.02 | (0.62; 6.59) | 0.70 | 0.60 | 0.245 |
|  | ^2^year_2011 | ↑ | 4.16 | (1.39; 12.45) | 1.43 | 0.56 | 0.011 |
|  | ^2^year_2012 |  | 1.39 | (0.25; 7.83) | 0.33 | 0.88 | 0.710 |
|  | herd_Lower Sabie |  | 1.74 | (0.79; 3.82) | 0.55 | 0.40 | 0.167 |
|  | herd_Mountain |  | 0.83 | (0.27; 2.56) | -0.19 | 0.58 | 0.745 |
|  | herd_Power Line | (↑) | 2.21 | (0.92; 5.29) | 0.79 | 0.45 | 0.076 |
|  | treatment |  | 1.01 | (0.58;1.75) | 0.01 | 0.28 | 0.974 |
|  | strongyle |  | 0.95 | (0.74; 1.23) | -0.05 | 0.13 | 0.699 |
|  | coccidia |  | 1.02 | (0.78; 1.34) | 0.02 | 0.14 | 0.860 |
|  | schisto | ↓ | 1.51 | (1.04; 2.20) | -0.41 | 0.19 | 0.030 |
|  | bTB |  | 0.87 | (0.47; 1.60) | -0.14 | 0.31 | 0.658 |
|  | brucella |  | 0.64 | (0.36; 1.15) | -0.45 | 0.30 | 0.135 |
| Lower | condition | ↓ |  |  | -0.59 | 0.28 | 0.032 |
| Sabie | age | ↓ |  |  | -0.02 | 0.01 | 0.037 |
|  | pregnant |  | 0.82 | (0.38; 1.81) | -0.19 | 0.40 | 0.630 |
|  | lactation | ↓ | 1.05 | (4.50; 19.28) | -1.50 | 0.74 | 0.043 |
|  | ^1^season_late wet |  | 0.42 | (0.14; 1.29) | -0.86 | 0.57 | 0.132 |
|  | ^1^season_early dry | ↓ | 1.13 | (2.73; 6.61) | -1.00 | 0.45 | 0.026 |
|  | ^1^season_late dry | ↓ | 15.95 | (2.00; 126.60) | -2.77 | 1.06 | 0.009 |
|  | ^2^year_2009 | ↑ | 6.23 | (1.37; 28.33) | 1.83 | 0.77 | 0.018 |
|  | ^2^year_2010 |  | 1.13 | (0.15; 8.29) | 0.12 | 1.02 | 0.907 |
|  | ^2^year_2011 | (↑) | 4.27 | (0.90; 20.35) | 1.45 | 0.80 | 0.068 |
|  | ^2^year_2012 |  | 2.44 | (0.20; 29.04) | 0.89 | 1.26 | 0.482 |
|  | treatment |  | 1.30 | (0.58; 2.92) | 0.27 | 0.41 | 0.519 |
|  | strongyle |  | 1.01 | (0.69; 1.47) | 0.01 | 0.19 | 0.975 |
|  | coccidia |  | 1.03 | (0.72; 1.48) | 0.03 | 0.18 | 0.862 |
|  | schisto |  | 0.69 | (0.39; 1.23) | -0.37 | 0.30 | 0.207 |
|  | bTB |  | 1.06 | (0.46; 2.45) | 0.06 | 0.43 | 0.895 |
|  | brucella |  | 0.69 | (0.29; 1.53) | -0.40 | 0.42 | 0.340 |
| Crocodile | condition |  |  |  | -0.46 | 0.32 | 0.147 |
| Bridge | age |  |  |  | -0.01 | 0.01 | 0.382 |
|  | pregnant |  | 1.07 | (0.49; 2.37) | 0.07 | 0.40 | 0.863 |
|  | lactation |  | 0.78 | (0.29; 2.65) | -0.25 | 0.63 | 0.690 |
|  | ^1^season_late wet |  | 1.14 | (0.38; 3.41) | 0.13 | 0.56 | 0.811 |
|  | ^1^season_early dry |  | 0.82 | (0.26; 2.59) | -0.20 | 0.59 | 0.737 |
|  | ^1^season_late dry |  | 1.25 | (0.48; 3.26) | 0.22 | 0.49 | 0.653 |
|  | ^2^year_2009 |  | 2.45 | (0.50; 12.16) | 0.90 | 0.82 | 0.271 |
|  | ^2^year_2010 |  | 2.52 | (0.52; 12.25) | 0.93 | 0.81 | 0.250 |
|  | ^2^year_2011 | (↑) | 4.09 | (0.88; 19.07) | 1.41 | 0.79 | 0.073 |
|  | ^2^year_2012 |  | 0.93 | (0.08; 10.57) | -0.07 | 1.24 | 0.952 |
|  | treatment |  | 0.79 | (0.37; 1.70) | -0.23 | 0.39 | 0.549 |
|  | strongyles |  | 0.93 | (0.66; 1.33) | -0.07 | 0.18 | 0.706 |
|  | coccidia |  | 0.96 | (0.63; 1.47) | -0.04 | 0.22 | 0.845 |
|  | schisto |  | 0.67 | (0.40; 1.12) | -0.40 | 0.26 | 0.128 |
|  | bTB |  | 0.69 | (0.27; 1.75) | -0.38 | 0.48 | 0.430 |
|  | brucella |  | 0.60 | (0.26; 1.40) | -0.51 | 0.43 | 0.238 |

^1^ Assessed using the residuals of condition on age due to high correlations between condition and age

^2^ Assessed as early wet, late wet, early dry and late dry

^3^ Assessed as 2008, 2009, 2010, 2011, 2012

**Table S5.** Correlation of fixed effects for the initial analysis included in models predicting dispersal likelihood of female African buffalo: Pooled data (923 observations total; 59 dispersal observations), Lower Sabie data (415 observations total; 30 dispersal observations), and Crocodile Bridge data (508 observations total; 29 dispersal observations). Model terms included in the correlation matrix include: condition (continuous), age (continuous), pregnancy status (binary), lactating status (binary), herd identification (categorical; only in pooled data), treatment (binary), schistosome infection (continuous), strongyle infection (continuous), coccidian infection (continuous), bTB status (binary), and brucellosis status (binary).

| Data | Fixed  effects | condition | age | pregnant | lactation | herd_ID | treatment | schisto | strongyle | coccidia | bTB | brucella |
| --- | --- | --- | --- | --- | --- | --- | --- | --- | --- | --- | --- | --- |
| Pooled | condition | - |  |  |  |  |  |  |  |  |  |  |
|  | age | -0.27 | - |  |  |  |  |  |  |  |  |  |
|  | pregnant | 0.01 | 0.23 | - |  |  |  |  |  |  |  |  |
|  | lactation | -0.17 | 0.37 | -0.15 | - |  |  |  |  |  |  |  |
|  | herd | -0.21 | 0.11 | 0.02 | -0.06 | - |  |  |  |  |  |  |
|  | treatment | 0.04 | 0.04 | -0.01 | 0.05 | -0.03 | - |  |  |  |  |  |
|  | schisto | -0.17 | 0.19 | 0.04 | 0.10 | 0.15 | 0.07 | - |  |  |  |  |
|  | strongyle | -0.10 | -0.20 | -0.11 | -0.03 | 0.06 | -0.22 | -0.03 | - |  |  |  |
|  | coccidia | 0.07 | -0.09 | -0.05 | 0.01 | -0.13 | -0.01 | -0.07 | 0.04 | - |  |  |
|  | bTB | -0.19 | 0.18 | 0.08 | 0.10 | 0.03 | 0.09 | -0.01 | 0.02 | 0.02 | - |  |
|  | brucella | -0.03 | 0.18 | 0.13 | 0.07 | -0.01 | 0.10 | 0.05 | -0.08 | -0.02 | 0.17 | - |
| Lower | condition | - |  |  |  |  |  |  |  |  |  |  |
| Sabie | age | -0.42 | - |  |  |  |  |  |  |  |  |  |
|  | pregnant | -0.14 | 0.17 | - |  |  |  |  |  |  |  |  |
|  | lactation | -0.22 | 0.41 | -0.18 | - |  |  |  |  |  |  |  |
|  | herd | NA | NA | NA | NA | - |  |  |  |  |  |  |
|  | treatment | 0.07 | 0.12 | -0.05 | 0.01 | NA | - |  |  |  |  |  |
|  | schisto | -0.17 | 0.34 | 0.11 | 0.18 | NA | 0.13 | - |  |  |  |  |
|  | strongyle | -0.05 | -0.22 | -0.10 | -0.01 | NA | -0.23 | -0.16 | - |  |  |  |
|  | coccidia | 0.00 | -0.08 | -0.02 | -0.08 | NA | -0.03 | 0.02 | 0.04 | - |  |  |
|  | bTB | -0.26 | 0.09 | 0.08 | 0.02 | NA | 0.14 | 0.07 | 0.05 | 0.03 | - |  |
|  | brucella | -0.03 | 0.11 | 0.20 | -0.01 | NA | 0.07 | 0.01 | -0.05 | 0.02 | 0.16 | - |
| Croc | condition | - |  |  |  |  |  |  |  |  |  |  |
| Bridge | age | -0.10 | - |  |  |  |  |  |  |  |  |  |
|  | pregnant | 0.13 | 0.28 | - |  |  |  |  |  |  |  |  |
|  | lactation | -0.21 | 0.34 | -0.16 | - |  |  |  |  |  |  |  |
|  | herd | NA | NA | NA | NA | - |  |  |  |  |  |  |
|  | treatment | 0.00 | -0.02 | 0.01 | 0.09 | NA | - |  |  |  |  |  |
|  | schisto | -0.08 | 0.08 | 0.03 | 0.10 | NA | 0.05 | - |  |  |  |  |
|  | strongyle | -0.07 | -0.20 | -0.10 | 0.01 | NA | -0.22 | -0.02 | - |  |  |  |
|  | coccidia | 0.05 | -0.09 | -0.11 | 0.06 | NA | -0.01 | -0.08 | 0.09 | - |  |  |
|  | bTB | -0.17 | 0.26 | 0.06 | 0.17 | NA | 0.04 | -0.04 | 0.02 | -0.02 | - |  |
|  | brucella | -0.06 | 0.25 | 0.07 | 0.14 | NA | 0.12 | 0.01 | -0.08 | -0.07 | 0.17 | - |

**Table S6.** Model selection of generalized linear mixed effect models from the secondary analyses predicting dispersal likelihood of female African buffalo (*Syncerus caffer*) in Kruger National Park, South Africa for the period June 2008 – August 2012, based on Akaike’s Information Criterion (AIC*_c_*) corrected for small sample size. We present the top 5 models for the pooled data (1349 observations total; 91 dispersal observations), Lower Sabie data (646 observations total; 52 dispersal observations), and Crocodile Bridge data (703 observations total; 39 dispersal observations). Model terms included in final models included condition (continuous), age (continuous), pregnancy and lactating status (binary), herd (categorical), season (categorical) and year (categorical). The AIC*_c_* weights presented are for all models.

| Data set | Models | K | AIC*_c_* | ∆AIC*_c_* | AIC*_c_*Wt |
| --- | --- | --- | --- | --- | --- |
| Pooled | condition+age+lactation+^2^year+herd | 13 | 627.98 | 0.00 | 0.34 |
|  | condition+age+^2^year+herd | 12 | 628.11 | 0.13 | 0.32 |
|  | condition+age+pregnant+lactation+^2^year+herd | 14 | 629.06 | 1.08 | 0.20 |
|  | condition+age+pregnant+^2^year+herd | 13 | 629.86 | 1.88 | 0.13 |
| Lower | age+pregnant+lactation+^1^season+^2^year | 13 | 327.24 | 0.00 | 0.33 |
| Sabie | age+pregnant+^1^season+^2^year | 12 | 327.47 | 0.24 | 0.29 |
|  | age+^1^season+^2^year | 11 | 327.90 | 0.67 | 0.23 |
|  | age+lactation+^1^season+^2^year | 12 | 328.81 | 1.58 | 0.15 |
| Crocodile | condition+age+^2^year | 9 | 298.65 | 0.00 | 0.56 |
| Bridge | condition+age+pregnant+^2^year | 10 | 300.39 | 1.74 | 0.23 |
|  | condition+age+lactation+^2^year | 10 | 300.62 | 1.97 | 0.21 |

^1^ Assessed as early wet, late wet, early dry and late dry

^2^ Assessed as 2008, 2009, 2010, 2011, 2012

**Table S7.** Univariate models of fixed effects for secondary analyses predicting dispersal likelihood of female African buffalo (Pooled data (1349 observations total; 91 dispersal observations), Lower Sabie data (646 observations total; 52 dispersal observations), and Crocodile Bridge data (703 observations total; 39 dispersal observations)). Univariates include condition (continuous), age (continuous), pregnancy and lactating status (binary), herd (categorical), season (categorical), year (categorical), with the nested random effect of (1|Animal_ID/Herd_ID) or random effect of Animal_ID to account for pseudoreplication and to acknowledge the different herds.

| Data set | Predictor | Effect on likelihood of dispersal | Odds-  ratio | Odds ratio (95% CIs) | Estimate | SE | p-value |
| --- | --- | --- | --- | --- | --- | --- | --- |
| Pooled | condition | ↓ |  |  | 0.25 | 0.16 | 0.130 |
|  | age | ↓ |  |  | -0.02 | 0.00 | <0.001 |
|  | pregnant |  | 0.73 | (0.45; 1.18) | -0.31 | 0.24 | 0.204 |
|  | lactation | ↓ | 2.40 | (1.14; 5.06) | -0.88 | 0.38 | 0.021 |
|  | ^1^season_late wet |  | 0.69 | (0.38; 1.26) | -0.37 | 0.19 | 0.225 |
|  | ^1^season_early dry | ↓ | 2.14 | (1.20; 3.82) | -0.76 | 0.30 | 0.010 |
|  | ^1^season_late dry | ↓ | 2.20 | (1.20; 4.01) | -0.79 | 0.31 | 0.010 |
|  | ^2^year_2009 | ↑ | 2.20 | (1.07; 4.54) | 0.79 | 0.37 | 0.032 |
|  | ^2^year_2010 |  | 1.35 | (0.59; 3.09) | 0.30 | 0.42 | 0.480 |
|  | ^2^year_2011 | ↑ | 2.40 | (1.12; 5.15) | 0.88 | 0.39 | 0.024 |
|  | ^2^year_2012 |  | 0.28 | (0.06; 1.31) | -1.27 | 0.78 | 0.107 |
|  | ^3^herd_Lower Sabie | ↑ | 2.36 | (1.20; 4.65) | 0.86 | 0.35 | 0.013 |
|  | ^3^herd_Mountain |  | 1.15 | (0.45; 2.95) | 0.14 | 0.48 | 0.768 |
|  | ^3^herd_Power Line | ↑ | 2.38 | (1.08; 5.24) | 0.87 | 0.40 | 0.031 |
| Lower | condition |  |  |  | -0.44 | 0.23 | 0.052 |
| Sabie | age | ↓ |  |  | -0.02 | 0.01 | <0.001 |
|  | pregnant | (↓) | 1.78 | (0.91; 3.50) | -0.58 | 0.34 | 0.092 |
|  | lactation | ↓ | 5.16 | (1.55; 17.11) | -1.64 | 0.61 | 0.007 |
|  | ^1^season_late wet | ↓ | 2.94 | (1.26; 6.86) | -1.08 | 0.43 | 0.013 |
|  | ^1^season_early dry | ↓ | 3.21 | (1.50; 6.88) | -1.17 | 0.39 | 0.003 |
|  | ^1^season_late dry | ↓ | 10.31 | (2.83; 37.59) | -2.33 | 0.66 | <0.001 |
|  | ^2^year_2009 | ↑ | 3.49 | (1.35; 9.03) | 1.25 | 0.48 | 0.010 |
|  | ^2^year_2010 |  | 1.04 | (0.27; 3.99) | 0.04 | 0.68 | 0.950 |
|  | ^2^year_2011 | (↑) | 2.58 | (0.89; 7.51) | 0.95 | 0.54 | 0.081 |
|  | ^2^year_2012 |  | 0.41 | (0.05; 3.61) | -0.89 | 1.11 | 0.422 |
| Crocodile | condition | (↓) |  |  | -0.53 | 0.27 | 0.054 |
| Bridge | age |  |  |  | -0.01 | 0.01 | 0.189 |
|  | pregnant |  | 1.02 | (0.51; 2.04) | 0.02 | 0.35 | 0.947 |
|  | lactation |  | 0.93 | (0.35; 2.47) | -0.07 | 0.50 | 0.890 |
|  | ^1^season_late wet |  | 1.78 | (0.63; 5.03) | 0.57 | 0.53 | 0.277 |
|  | ^1^season_early dry |  | 2.18 | (0.83; 5.75) | 0.78 | 0.50 | 0.115 |
|  | ^1^season_late dry |  | 2.32 | (0.77; 6.96) | 0.84 | 0.56 | 0.133 |
|  | ^2^year_2009 |  | 1.16 | (0.35; 3.86) | 0.15 | 0.61 | 0.808 |
|  | ^2^year_2010 |  | 1.80 | (0.56; 5.80) | 0.59 | 0.60 | 0.327 |
|  | ^2^year_2011 | (↑) | 2.79 | (0.89; 8.74) | 1.03 | 0.58 | 0.079 |
|  | ^2^year_2012 |  | 0.24 | (0.03; 2.17) | -1.44 | 1.13 | 0.203 |

^1^ Assessed as early wet, late wet, early dry and late dry

^2^ Assessed as 2008, 2009, 2010, 2011, 2012

**Table S8.** Correlation matrix of fixed effects for the secondary analysis included in models predicting dispersal likelihood of female African buffalo: Pooled data (1349 observations total; 91 dispersal observations), Lower Sabie data (646 observations total; 52 dispersal observations), and Crocodile Bridge data (703 observations total; 39 dispersal observations). Model terms included in the correlation matrix include condition (continuous), age (continuous), pregnancy status (binary), lactating status (binary), and herd identification (categorical; only in pooled data).

| Data | Fixed effects | condition | age | pregnant | lactation |
| --- | --- | --- | --- | --- | --- |
| Pooled | condition |  |  |  |  |
|  | age | -0.28 |  |  |  |
|  | pregnant | -0.01 | 0.27 |  |  |
|  | lactation | -0.19 | 0.38 | -0.09 |  |
|  | herd | -0.22 | 0.15 | 0.05 | -0.05 |
| Lower | condition |  |  |  |  |
| Sabie | age | -0.41 |  |  |  |
|  | pregnant | -0.14 | 0.23 |  |  |
|  | lactation | -0.25 | 0.43 | -0.11 |  |
| Croc | condition |  |  |  |  |
| Bridge | age | -0.13 |  |  |  |
|  | pregnant | 0.10 | 0.33 |  |  |
|  | lactation | -0.23 | 0.34 | -0.10 |  |
